# Lung function in idiopathic pulmonary fibrosis - extended analyses of the IFIGENIA trial

**DOI:** 10.1186/1465-9921-10-101

**Published:** 2009-10-27

**Authors:** Jürgen Behr, Maurits Demedts, Roland Buhl, Ulrich Costabel, Richard PN Dekhuijzen, Henk M Jansen, William MacNee, Michiel Thomeer, Benoit Wallaert, Francois Laurent, Andrew G Nicholson, Eric K Verbeken, Johny Verschakelen, CDR Flower, Stefano Petruzzelli, Paul De Vuyst, JMM van den Bosch, Eulogio Rodriguez-Becerra, Ida Lankhorst, Marco Sardina, Gabrielle Boissard, the Ifigenia study group*

**Affiliations:** 1 Medizinische Klinik I, Klinikum Grosshadern der Ludwig Maximilians-Universität, München, Germany; 2 University Hospital, Katholieke Universiteit Leuven, Belgium; 3 III Medizinische Klinik, Klinikum der Johannes Gutenberg-Universität, Mainz, Germany; 4 Medical Faculty University of Duisburg-Essen and Ruhrlandklinik, Essen-Heidhausen, Germany; 5 University Medical Centre Nijmegen, the Netherlands; 6 Academic Medical Centre, Amsterdam, the Netherlands; 7 University of Edinburgh Medical School, Edinburgh, UK; 8 CHRU de Lille, Hôpital Calmette, Lille, France; 9 Hâopital Cardiologique, CHU de Bordeaux, France; 10 Royal Brompton Hospital, UK; 11 Evelyn Hospital, Cambridge, UK; 12 Dipartimento Cardio-Toracico, Università degli Studi di Pisa, Italy; 13 Université Libre de Bruxelles, Erasmus Hospital, Brussels, Belgium; 14 St Antonius Ziekenhuis Nieuwegein, the Netherlands; 15 Hospital Universitario Virgen del Rocio, Sevilla, Spain; 16 Zambon Group, Bresso, Milan, Italy

## Abstract

**Background:**

The randomized placebo-controlled IFIGENIA-trial demonstrated that therapy with high-dose N-acetylcysteine (NAC) given for one year, added to prednisone and azathioprine, significantly ameliorates (i.e. slows down) disease progression in terms of vital capacity (VC) (+9%) and diffusing capacity (DLco) (+24%) in idiopathic pulmonary fibrosis (IPF). To better understand the clinical implications of these findings we performed additional, explorative analyses of the IFGENIA data set.

**Methods:**

We analysed effects of NAC on VC, DLco, a composite physiologic index (CPI), and mortality in the 155 study-patients.

**Results:**

In trial completers the functional indices did not change significantly with NAC, whereas most indices deteriorated with placebo; in non-completers the majority of indices worsened but decline was generally less pronounced in most indices with NAC than with placebo. Most categorical analyses of VC, DLco and CPI also showed favourable changes with NAC. The effects of NAC on VC, DLco and CPI were significantly better if the baseline CPI was 50 points or lower.

**Conclusion:**

This descriptive analysis confirms and extends the favourable effects of NAC on lung function in IPF and emphasizes the usefulness of VC, DLco, and the CPI for the evaluation of a therapeutic effect. Most importantly, less progressed disease as indicated by a CPI of 50 points or lower at baseline was more responsive to therapy in this study.

**Trial Registration:**

Registered at http://www.ClinicalTrials.gov; number NCT00639496.

## Introduction

Idiopathic pulmonary fibrosis (IPF) is a distinct clinical and histopathologic entity, which accounts for approximately two thirds of the idiopathic interstitial pneumonias (IIP), and is associated with a high median mortality of 50% within 3 to 5 years [1-5]. The cause of this disease is unknown [4,6]. In the absence of a satisfactory treatment - except for lung transplantation in selected patients - the joint consensus statement on IPF by the American Thoracic Society and the European Respiratory Society suggested prednisone plus azathioprine (or cyclophosphamide) as potential treatment for IPF [4]. Based on this statement the recently published IFIGENIA trial used prednisone plus azathioprine as a standard treatment for all patients who were randomly assigned to receive additionally high-dose N-acetylcysteine (NAC) or placebo [7]. A treatment effect in favour of NAC was observed with a statistically significant smaller decrease of vital capacity (VC) (9%) and diffusing capacity (DLco) (24%) [7], which was in line with a previous observation [8]. However, in view of a drop-out rate of ca. 30% (including deaths) questions have been raised regarding the clinical relevance and robustness of the treatment effect [9]. These appropriate questions prompted us to perform additional, exploratory analyses of the data set. We evaluated subgroups of completers versus non-completers for differences in treatment response. We also calculated the Composite Physiologic Index (CPI), which uses the individual values for VC (% pred.), DLco (% pred.) and FEV1 (% pred.) to calculate the extent of fibrosis according to an empirically developed equation [10]. Based on low or high baseline CPI we compared treatment effects on patients with less progressed or progressed disease. We think that these additional exploratory analyses of the IFIGENIA data set offer a unique opportunity to provide insight into the clinical course of the disease, to describe the potential clinical benefit of antioxidant therapy using NAC, and to explore the influence of disease severity at baseline on outcome.

## Methods

### Overall Design

Using data from the IFIGENIA trial [7], we performed a series of exploratory analyses of physiologic variables and characteristics in patients receiving prednisone and azathioprine as suggested by the ATS/ERS Statement [4] plus either high-dose NAC or placebo. The IFIGENIA study was a multinational, double-blind, randomized, placebo-controlled, parallel-group trial. Study treatment consisted of NAC (Zambon Group) 600 mg effervescent tablets t.i.d. or matched placebo. Patients (18 to 75 years) with a histological or radiological pattern typical for usual interstitial pneumonia (UIP) [1,5] were included after active exclusion of other diseases [4,7]. Patients were excluded in whom the standard regimen with prednisone and azathioprine was contraindicated or not justified (i.e. in stable patients) (full in- and exclusion criteria see reference [7]).

The study was conducted according to GCP-ICH guidelines goverend by the Declaration of Helsinki and by national regulations. The protocol was approved by the local ethics committees. All patients gave written informed consent and were free to withdraw at any time. Regular monitoring and sample audits were performed at the centers throughout the study. The study was designed and analysed by 19 academic physicians experienced in idiopathic pulmonary fibrosis, 1 independent statistician, and three representatives of the sponsor. The sponsor held the data but placed no limitations on study design, data analysis, or the content of the manuscript. The main statistical analysis was performed by an independent statistical company (Innopharma, Milan, Italy).

### Setting, Participants, and Randomization

Between March 2000 and July 2002 184 patients were screened in 36 centers from six countries. Out of 182 eligible patients twenty-seven were not included in the analysis: 5 patients withdrew consent before starting treatment and 22 (12%) were excluded because the IPF-diagnosis was not confirmed by the radiology or histology expert committee [7]. Of the 155 patients included in the analysis, 80 patients had been randomized to NAC and 75 to placebo. There were no significant differences in baseline characteristics [7].

Of these 155 patients, 108 (70%) completed the one year study (57 on NAC, 51 on placebo). At baseline 8 patients on NAC and 2 on placebo used long-term oxygen therapy (LTOT); during the study, 3 additional patients on NAC and 11 on placebo were started on LTOT. Forty-seven patients (30%) did not complete the one year study: 32 patients (21%) withdrew for various reasons (16 on NAC and 16 on placebo) and 15 (10%) died during the study: 7 (9%) on NAC, and 8 (11%) on placebo (p = 0.69).

### Measurements

Vital capacity (VC), forced expired volume in 1 sec (FEV_1_) and single-breath CO-diffusing capacity (DLco) were measured and cardiopulmonary exercise testing was performed according to ERS guidelines [11,12].

The composite physiologic index (CPI) was calculated [10]: CPI = 91.0 - 0.65 × DLco(%pred) -0.53 VC(%pred) + 0.34 FEV_1_(%pred). CPI is in-between 20 and 80 points, higher points indicating more fibrosis and poorer prognosis.

### Statistical Analysis

As in the previous publication [7] the main statistical analysis was based on a stepwise, fixed-effects analysis of covariance using an iterative procedure including the change from baseline as response variable, treatment and country factors as fixed effects, baseline value as covariate and following potential cofactors: smoking history (current/ex-smokers vs. never-smokers), age (<65 years vs. ≥ 65 years), duration of disease since diagnosis (≤ 6 vs. >6 months), DLco(≤ 40% vs. >40% predicted), VC (≤ 60% vs. >60% predicted), sex (male vs. female), and whether a biopsy had been performed. This former model was also used for the analysis of the composite physiologic index (CPI). For the analysis of CPI changes from baseline, according to categorised baseline CPI, an ANOVA model was used, including treatment and baseline CPI category as fixed factors (basal CPI: ≤ 50; >50) and their interaction; the cut-off value of 50 for basal CPI approximated the median of baseline CPI (52.26). Using the median itself did not impact on the results. Changing CPI cut-offs by steps of one and five points, respectively, turned out to show that a CPI of 50 points as cut-off appeared to be optimal.

The analyses were based, as for the main analysis, on data from all randomized patients who met the inclusion criteria for the study, received the trial medication at least once, and underwent at least baseline observation. Missing data were replaced by the last observation-carried-forward (LOCF) method for all patients who underwent at least one lung-function measurement after baseline [7]. As the data were reanalysed minor changes from the original results occurred based on differences in adjustments with baseline and covariates as well as rounding during calculations. As a descriptive analysis the course of completers and non-completers was evaluated with respect to treatment arm; the completer subset included all patients with an assessment available at the 12 months final visit, whereas non-completers were either drop-outs or patients with a missing 12 months assessment.

Cardio-pulmonary exercise test derived variables were analysed including only treatment as fixed effect and the baseline value as a covariate.

For categorical analysis of lung function indices using variable cut-off values, comparisons between NAC and placebo were performed using 2-sided Fisher exact tests. No adjustment for multiple testing was performed, these analyses being considered as exploratory.

## Results

### Analysis of VC, DLco, and CPI: last-observation-carried-forward (LOCF) vs. Completers and Non-Completers

For the analysis of completers and non-completers (table 1 and 2) both groups did not differ significantly in demographic characteristics, but as could be expected, lung function variables, PaO_2 _and CPI were worse in the non-completers (table 1). By definition, the exposition to study medication was 12 months in completers and shorter in non-completers (5.4 ± 2.5 months). An individual analysis of the four data subsets (i.e. completers NAC/Placebo and non-completers NAC/Placebo) revealed significant declines in VC (l) and in DLco (mmol/min/kPa and % predicted) in completers and non-completers with placebo, whereas with NAC VC (l and % pred.) stayed stable in both completers and non-completers (table 2). With NAC DLco (mmol/min/kPa and % pred.) remained unchanged in completers and declined in the non-completers (table 2).

**Table 1:** Baseline Characteristics of Completers and non-Completers:

		Completers (N = 106)	Non completers (N = 33)	p value
**All patients [n(%)]**		106 (100.0%)	33 (100.0%)	
**Gender [n(%)]**	Male	74 (69.8%)	25 (75.8%)	0.5100
	Female	32 (30.2%)	8 (24.2%)	
**Age (Year)**		62.1 (9.0)	64.2 (8.3)	0.2219
**Duration of symptoms (Month)**		42.6 (44.4)	35.1 (34.9)	0.3812
**Pretreatment [n(%)]**	No	52 (49.1%)	17 (51.5%)	0.8052
	Yes	54 (50.9%)	16 (48.5%)	
**New diagnosis? [n(%)]**	No	71 (67.0%)	16 (48.5%)	0.0552
	Yes	35 (33.0%)	17 (51.5%)	
**Surgical lung biopsy [n(%)]**	No	59 (55.7)	16 (48.5%)	0.1794
	Yes	47 (44.3%)	17 (51.5%)	
**Baseline VC [l]**		2.38 (0.72)	2.14 (0.65)	0.0901
**Baseline DLCo [mmol/min/kPa]**		4.03 (1.33)	3.07 (1.34)	0.0004 ***
**Baseline TLC [l]**		3.78 (0.98)	3.34 (0.89)	0.0261 *
**Baseline PaO_2 _[mmHg]**		73.23 (11.11)	66.98 (13.66)	0.0093 **
**Baseline P(A-a)O_2 _[mmHg]**		30.79 (13.82)	38.88 (25.17)	0.0376 *
**Baseline FEV1/VC**		0.840 (0.081)	0.841 (0.076)	0.9026
**Baseline CPI**		49.20 (10.71)	57.00 (9.75)	0.0003 ***

*: p < 0.05; **: p < 0.01; ***: p < 0.001;

**Table 2:** LOCF-method vs. Completers/non-Completers analyses of VC, DLco, and CPI: changes between last measurement and base line

	LOCF-method		Completers		Non-Completers	
Change from baseline †	NAC	PLA	NAC	PLA	NAC	PLA
VC (l)	-0.057	-0.233	-0.062	-0.148	-0.207	-0.424
± SE	± 0.054	± 0.057	± 0.063	± 0.069	± 0.114	± 0.119
n =	71	68	55	51	16	17
p =	0.29	<0.0001	0.33	0.036	0.084	0.0017
VC (% pred.)	-0.830	-5.634	-1.245	-3.178	-2.849	-10.109
± SE	± 1.426	± 1.539	± 1.655	± 1.837	± 3.241	± 3.590
n =	71	68	55	51	16	17
p =	0.56	0.0004	0.434	0.087	0.39	0.0098
DLco (mmol/min/kPa)	-0.116	-0.652	0.339	-0.767	-0.832	-1.212
± SE	± 0.226	± 0.226	± 0.295	± 0.322	± 0.279	± 0.222
n =	68	63	48	46	20	17
p =	0.61	0.0047	0.25	0.02	0.0063	<0.0001
DLco(% pred.)	-2.124	-7.361	-0.697	-7.801	-8.288	-11.861
± SE	± 1.809	± 1.790	± 2.239	± 2.231	± 3.034	± 2.580
n =	68	63	48	46	20	17
p =	0.24	<0.0001	0.76	0.0008	0.011	<0.0001
CPI	0.509	5.471	-1.086	5.065	6.854	9.867
± SE	± 1.474	± 1.459	± 1.812	± 1.810	± 2.488	± 2.118
n =	68	64	47	46	21	18
p =	0.73	0.0003	0.55	0.0065	0.010	<0.0001

† Least Square Means, relative SE and within groups p values were calculated from the model described in method

When formally assessing the differences between treatment arms (ANCOVA model), a significant effect on VC of 0.18 ± 0.07 L and 4.80 ± 2.00% pred. was found when using the LOCF method (p = 0.017). For the completers the effect was smaller (0.09 ± 0.09 L, p = 0.34 and 1.93 ± 2.41% pred., p = 0.42) and more pronounced in non-completers (0.22 ± 0.13 L, p = 0.099 and 7.26 ± 3.80% pred., p = 0.069). For the DLco measurements NAC had a statistically significant treatment effect in the completer subset for absolute change and for % predicted (1.106 ± 0.377 mmol/min/kPa, p = 0.0044 and 7.10 ± 2.64% pred., p = 0.0087, respectively), but not so in the small non-completer subset (0.380 ± 0.228 mmol/min/kPa, p = 0.11 and 3.573 ± 2.764% pred., p = 0.21).

We also calculated and analysed the Composite Physiological Index (CPI) [10]. At baseline CPI mean ± SD was similar in NAC vs. placebo treated patients (52.6 ± 9,8 vs. 50.93 ± 10.2, n.s.). An increase of the CPI indicates disease progression and was observed in the placebo group using the LOCF method and also in the completer and non-completer subsets using placebo, whereas NAC-treated patients did not show significant disease progression in terms of the CPI in the LOCF and completer subsets (table 2). In the non-completers disease progression occurred with both, NAC (p = 0.010) and placebo (p < 0.0001)(table 2). Again, the formal analysis of the treatment effect between groups (ANCOVA model) was significant in favour of NAC when using the LOCF method (-4.962 ± 1.607, p = 0.0025) and in the completer subset (-6.151 ± 2.137, p = 0.0052); in the smaller non-completer subset the same trend occurred (-3.014 ± 2.201, p = 0.20).

### Analysis of cardio pulmonary exercise testing: LOCF vs. Completers and Non-Completers

Using the LOCF method and also in the completer subgroup, significant declines of W'max, V'CO_2_max and V'O_2 _max during exercise were found with placebo, whereas no significant changes occurred with NAC (table 3). The difference between the NAC and placebo groups was statistically significant in favour of NAC for V'CO_2_max (p = 0.033) using the LOCF method, and was statistically significant favouring NAC for V'CO_2_max, V'O_2_max and V'O_2_max % pred in the completer subset. The non-completer group was small for placebo (n ≤ 9) and NAC (n ≤ 7) (table 3) and showed no significant differences.

**Table 3:** LOCF-method vs. Completers/non-Completers analyses of cardio pulmonary exercise test derived variables: changes between last measurement and baseline

	LOCF-method		Completers		Non-Completers	
	NAC	PLA	NAC	PLA	NAC	PLA
W'max (W)	-3.693	-11.136	-1.259	-11.183	-19.262	-10.826
± SE	± 3.534	± 3.382	± 3.90	± 3.849	± 6.056	± 4.944
n =	44	48	38	39	6	9
p =	0.30	0.0014	0.75	0.0048	0.0079	0.0049
V'CO_2_max (L/min)	0.035	-0.138	0.0833	-0.141	-0.207	-0.128
± SE	± 0.0579	± 0.0548	± 0.0632	± 0.0599	± 0.127	± 0.118
n =	43	48	36	40	7	8
p =	0.55	0.014	0.19	0.022	0.13	0.30
V'O_2_max (L/min)	0.004	-0.121	0.0833	-0.141	-0.231	-0.105
± SE	± 0.050	± 0.048	± 0.056	0.054	0.108	0.095
n =	44	48	37	39	7	9
p =	0.93	0.014	0.39	0.025	0.051	0.29
V'O_2_max (% pred.)	-2.411	-8.628	-0.950	-9.473	-10.090	-4.993
± SE	± 2.554	± 2.445	± 2.786	± 2.713	± 5.180	± 4.562
n =	44	48	37	39	7	9
p =	0.35	0.0007	0.73	0.0008	0.073	0.29

### Categorical analysis of VC, DLco and CPI and effect of baseline value of CPI

**Figure 1:**
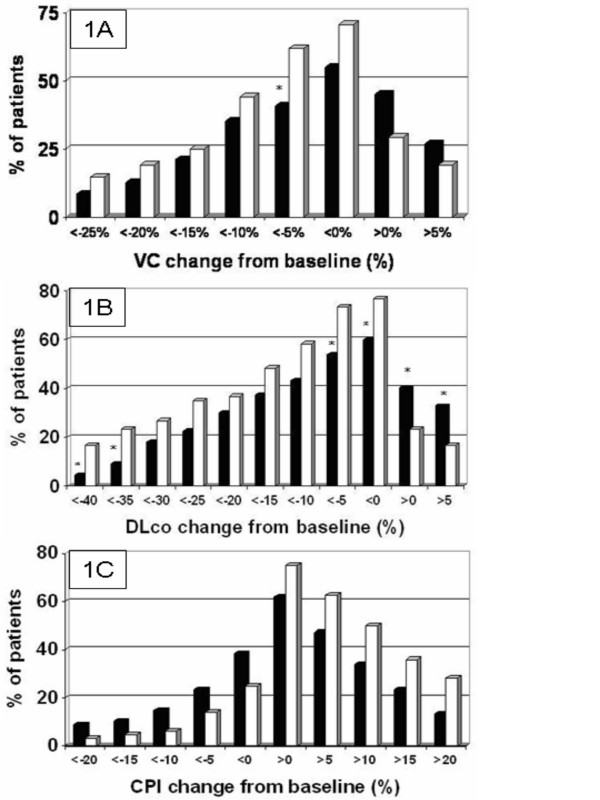
**Categorical analyses in steps of 5% change from baseline during the one year study period**. **a: VC: **Higher percentages of patients receiving NAC (black columns) showed any improvement and improvement of 5% or more as compared to baseline, whereas higher percentages of patients receiving placebo (white columns) showed any deterioration or decreases of VC of more than 5, 10, 15, 20, and 25% from baseline; this difference was significant at the 5% level (* p < 0.05 NAC vs plac). **b: DLco**^#^: A higher percentage of patients receiving NAC (black columns) showed improvement and a lower percentage of patients receiving NAC (black columns) showed deterioration of DLco as compared to placebo (white columns). The differences were statistically significant at the levels <-40%, <-35%, <-5%, >5%, any deterioration, and any improvement (* p < 0.05 NAC vs plac for each of these comparisons). ^# ^numbers refer to DLco Hb corrected. **c: CPI: **A higher percentage of patients receiving NAC (black columns) showed improvement (i.e. decrease) and a lower percentage of patients receiving NAC (black columns) showed deterioration (i.e. increase) of the CPI as compared to placebo (white columns). The differences were statistically not significant.

Categorical frequency analyses were performed at 5% intervals for the changes in VC, DLco and CPI, to evaluate how many patients would have achieved a specific predefined endpoint. With NAC therapy significantly less patients suffered a 5% or more deterioration of VC from baseline as compared to placebo (40.8 vs. 61.8%, p = 0.018) (fig. 1A). Similarly, less patients with NAC therapy as compared to placebo deteriorated with respect to DLco at several levels: with any deterioration (59.7% vs. 76.7%, p = 0.057), with more than 5% deterioration (53.7% vs. 73.3%, p = 0.028), with more than 35% deterioration (9.0% vs. 23.3%, p = 0.030), and with more than 40% deterioration (4.5% vs. 16.7%, p = 0.037) (fig. 1B). Moreover, a higher proportion of patients had improvements in their DLco with NAC as compared to placebo at the level of any improvement (40.3% vs. 23.3%, p < 0.05) and with an improvement greater than 5% from baseline (32.8% vs. 16.7%, p = 0.042). For CPI a similar trend was observed.

### Influence of baseline CPI on outcome

**Figure 2:**
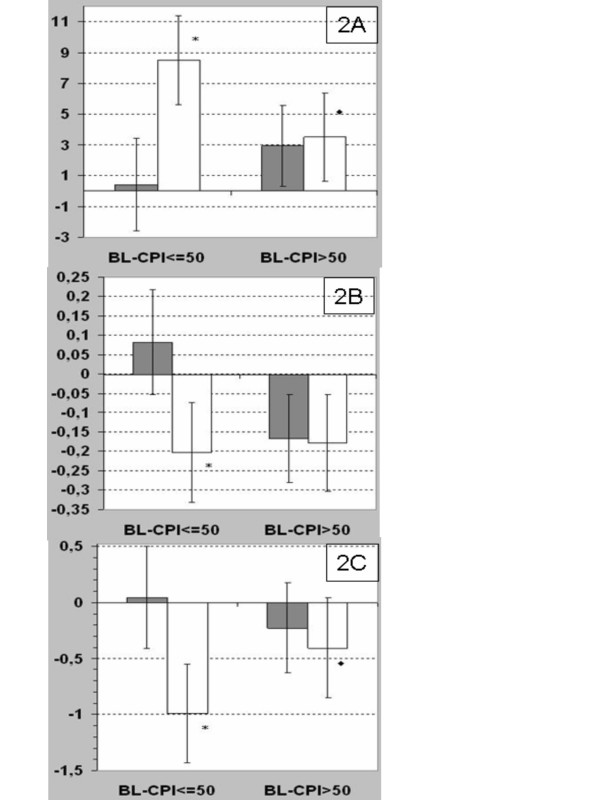
**Effects of NAC on CPI, VC, and DLco, depending on baseline CPI being lower or higher than 50 points**. **a: **Effect of NAC therapy on change in CPI from baseline, depending on baseline CPI being lower or higher than 50 points. Black columns = NAC; white columns = placebo. *LS means and 95% confidence interval for changes from baseline in a model including treatment and CPI category as fixed factors are plotted*. * p = 0.0002 vs BL-CPI< = 50 NAC. ◆ p = 0.016 vs. BL-CPI< = 50 Placebo **b: **Effect of NAC therapy on change in VC from baseline, depending on baseline CPI being lower or higher than 50 points. Black columns = NAC; white columns = placebo. *LS means and 95% confidence interval for changes from baseline in a model including treatment and CPI category as fixed factors are plotted*. * p = 0.0031 vs BL-CPI< = 50 NAC. p = 0.0066 between both NAC subunits. **c: **Effect of NAC therapy on change in DLco from baseline, depending on baseline CPI being lower or higher than 50 points. Black columns = NAC; white columns = placebo. *LS means and 95% confidence interval for changes from baseline in a model including treatment and CPI category as fixed factors are plotted*. * p = 0.0015 vs BL-CPI< = 50 NAC. ◆ p = 0.067 vs. BL-CPI< = 50 Placebo.

The baseline CPI influenced the outcome of therapy: the patients with less advanced disease as indicated by a baseline CPI ≤ 50 showed effects favouring NAC for changes of the CPI itself (net effect after one year 8.11 points, p = 0.0002), the VC (net effect after one year 0.285 l, p = 0.0031), and the DLco (net effect after one year 1.042 mmol/min/kPa, p = 0.0015). In contrast, only small, statistically not significant trends (favouring NAC) were observed in patients with a baseline CPI > 50 points (ΔCPI 0.560 points, n.s.; ΔVC 0.012 l, n.s.; ΔDLco 0.177 mmol/min/kPa, n.s.). (See figures 2a-c).

### Analysis of patients who survived/died

Of the total population of 155 patients 15 (10%) died; of these 7 (9%) were in the NAC arm and 8 (11%) in the placebo arm. Only four out of 15 patients who died (26.7%) had significant deterioration of PFT as defined in the ATS/ERS-statement [4] prior to death, whereas eight (53.3%) died within three months of treatment initiation without follow-up PFTs; 4 of these latter 8 patients died from cardiac disease, 3 succumbed to overwhelming infection, and one died from respiratory failure. Analysis of patients who survived or died with NAC or placebo showed no statistically significant differences in baseline demographics between the four groups. However, P(A-a)O_2 _and CPI were significantly higher at baseline in those patients who died during the study (data not shown).

## Discussion

This study extends the analysis of our previously published IFIGENIA study [7] by categorical analysis of lung function indices and description of completer/non-completer subsets. In addition we analysed the composite physiologic index (CPI) which was especially designed and validated to represent disease extent on HRCT and to correct for coexisting emphysema and which has also been shown to be a predictor of prognosis [10,13]. The CPI has advantages over scoring system as proposed by Watters et al [14] and by King et al. [15] because it is easier to generate, requiring solely the measurement of VC, FEV_1 _and DLco, and predicts survival more consistently [10,13].

When analysing the effects of NAC on VC, DLco, maximal exercise indices, and the CPI we found that in completers none of the indices deteriorated significantly with NAC, but most indices did with placebo; in non-completers the majority of indices worsened at a much lesser extent with NAC than with placebo. Importantly, we did not observe divergent signals from the completers and non-completers subgroups regarding treatment effects. Correspondingly, categorical changes in VC, DLco and CPI showed globally significantly better results with NAC than with placebo.

Our data indicate that the effects of NAC on VC, DLco and CPI were significantly better if the baseline CPI was low (≤ 50 points), i.e. in the less severe cases. This finding is in line with findings from previous studies and may suggest the presence of specific disease processes in severe disease states [15-18].

With respect to the clinical relevance of the changes observed several authors have demonstrated in IPF cohorts that small changes of FVC or DLco during the first 6 to12 months eventually result in major survival differences during the following years [13,19-22]. Consequently, small treatment effects achieved with high dose NAC therapy may impact survival significantly in the long run [23]. A long-term clinical trial testing NAC in IPF is clearly warranted.

Regarding IPF mortality it should be noted that lung function deterioration could be documented before death only in a minority of patients (26,7%), whereas the majority of patients (53.3%) died with rapid deterioration within three months of treatment not allowing for control lung function measurements before death. This may imply that a decline of lung function does not precede a fatal disease exacerbation, as hypothesized previously [22] or simply that the intervals at which control measurements of lung function were scheduled were too long. This should be considered in future studies. A considerable number of patients died from coexisting cardiac disease. This observation should draw attention to potential comorbidities like coronary artery and left heart diseases as well as pulmonary hypertension associated with IPF which may warrant specific treatment approaches [24].

There are limitations of our study. The completers/non-completers groups used for comparisons do not represent predefined and stratified subgroups. Therefore, the groups differ e.g. with respect to baseline lung function. On the other hand it is not surprising and reassuring that the non-completer group presented worse baseline values. The explorative statistical analysis presented here was done without correction for multiple testing, thus limiting its use for clinical decision making. Moreover, our data do not allow firm conclusions to be drawn on whether the treatment effects observed are contributable to NAC alone or can be achieved only when using triple therapy of prednisone, azathioprine, and high-dose NAC. Finally, it may be argued that intention to treat is the only acceptable study design; although we do not question this dictum for the pivotal analysis, additional exploratory analyses, as the one presented here, may allow a better understanding of the data structure and robustness and may help to generate new hypothesis and should, therefore, also be made accessible.

## Conclusion

Taken together the data presented here corroborate the results of the pivotal paper [7] and support the use of high-dose NAC in patients with IPF. Although the effect size appears relatively small, the lack of serious side effects allows a positive risk-to-benefit assessment. Notably, our present analysis suggests that patients with less progressed disease (CPI ≤ 50 points) may have a more favourable response. Consequently, "triple therapy" - prednisone, azathioprine plus high-dose NAC - is a seizable treatment option for IPF patients. Referring to the available evidence the recent ILD Guideline published by the British Thoracic Society incorporated a weak recommendation (C) in favour of high-dose NAC in combination with prednisone and azathioprine for IPF patients [25].

## Competing interests

The authors declare that they have no competing interests. JB, MD, RB, UC, PNRD, HMJ, WMN, MT, and BW were members of the steering committee and made significant conceptual and intellectual contributions to the design and conception of the study. FL, AGN, EKV, JV, and CDRF performed central reading of histology slides and HRCT scans, respectively, to confirm the diagnosis. SP, PDV, JMMvdB, ERB, IL, and MS participated in the study design and coordination and helped to draft the manuscript. GB performed the statistical analysis. All authors read and approved the final manuscript.

## Authors' contributions

All authors listed made significant conceptual and intellectual contributions to the design and conception of the study, substantially contributed to the article, and have provided final approval of the version submitted.
